# Prevalence and impact of alcohol and other drug use disorders on sedation and mechanical ventilation: a retrospective study

**DOI:** 10.1186/1471-2253-7-3

**Published:** 2007-03-14

**Authors:** Marjolein de Wit, Sau Yin Wan, Sujoy Gill, Wendy I Jenvey, Al M Best, Judith Tomlinson, Michael F Weaver

**Affiliations:** 1 Virginia Commonwealth University, Department of Internal Medicine, Richmond, Virginia, USA; 2 Virginia Commonwealth University, Division of Pulmonary and Critical Care Medicine, Richmond, Virginia, USA; 3 University of Miami, Department of Medicine, Division of Pulmonary and Critical Care Medicine, Miami, Florida, USA; 4 Virginia Commonwealth University, Department of Biostatistics, Richmond, Virginia, USA; 5 Virginia Commonwealth University, Department of Psychiatry, Division of Addiction Psychiatry, Richmond, Virginia, USA

## Abstract

**Background:**

Experience suggests that patients with alcohol and other drug use disorders (AOD) are commonly cared for in our intensive care units (ICU's) and require more sedation. We sought to determine the impact of AOD on sedation requirement and mechanical ventilation (MV) duration.

**Methods:**

Retrospective review of randomly selected records of adult patients undergoing MV in the medical ICU. Diagnoses of AOD were identified using strict criteria in Diagnostic and Statistical Manual of Mental Disorders, and through review of medical records and toxicology results.

**Results:**

Of the 70 MV patients reviewed, 27 had AOD (39%). Implicated substances were alcohol in 22 patients, cocaine in 5, heroin in 2, opioids in 2, marijuana in 2. There was no difference between AOD and non-AOD patients in age, race, or reason for MV, but patients with AOD were more likely to be male (21 versus 15, p < 0.0001) and had a lower mean Acute Physiology and Chronic Health Evaluation II (22 versus 26, p = 0.048). While AOD patients received more lorazepam equivalents (0.5 versus 0.2 mg/kg.day, p = 0.004), morphine equivalents (0.5 versus 0.1 mg/kg.day, p = 0.03) and longer duration of infusions (16 versus 10 hours/day. medication, p = 0.002), they had similar sedation levels (Richmond Agitation-Sedation Scale (RASS) -2 versus -2, p = 0.83), incidence of agitation (RASS ≥ 3: 3.0% versus 2.4% of observations, p = 0.33), and duration of MV (3.6 versus 3.9 days, p = 0.89) as those without AOD.

**Conclusion:**

The prevalence of AOD among medical ICU patients undergoing MV is high. Patients with AOD receive higher doses of sedation than their non-AOD counterparts to achieve similar RASS scores but do not undergo longer duration of MV.

## Background

Sedative and opioid agents are routinely administered to critically ill patients to treat agitation and facilitate mechanical ventilation (MV) [1]. Appropriate use of these agents is important as severe agitation is associated with prolonged MV and increased risk of self-extubation [2]. Excessive sedation administration is also associated with prolonged MV, and strategies aimed to limit oversedation have been found to decrease MV duration [3-7].

Alcohol and other drug use disorders (AOD) affect 9.4% of the American population, and prevalence of these disorders in intensive care units (ICU's) ranges from 5 to 30% [8-11]. Unlike patients without AOD, evidence suggests that patients with AOD on MV may develop withdrawal syndromes if undersedated or with early withdrawal of sedation, and sedative agents have been found to reduce the duration of alcohol withdrawal delirium [12,13]. However, the sedative requirements of patients with AOD have not been studied extensively.

Because there has been an increased focus recently on minimizing sedation to improve MV outcomes, and because patients with AOD may require a different approach to sedation while on MV, we designed a study to determine the prevalence of AOD and sedation needs among our medical ICU patients undergoing MV. We hypothesized that patients with AOD would require higher doses of sedatives and opioids, have more episodes of agitation, and require a longer duration of MV than those without AOD. The results of this study have previously been published in abstract form [14].

## Methods

The study was conducted in accordance with the ethical standards of the Virginia Commonwealth University's Office of Research Subject Protection and the Declaration of Helsinki of 1975, as revised in 1983. The study was approved by Virginia Commonwealth University Office of Research Subject Protection, Richmond, Virginia, and the need for consent was waived. The study was a retrospective cohort study of patient medical records. Medical patients admitted to our medical ICU who required invasive MV were eligible for study participation. The medical ICU is a closed unit where patients have similar surroundings. All beds are located in close proximity to nursing stations and medical equipment. When monitoring equipment alarms, the alarm not only sounds at the nursing stations but also in all patient rooms. Only patients physically located in the medical ICU were eligible for study participation thereby assuring that the noise exposure was similar for all study patients.

Using a random number generator, patients were selected from a list of all patients undergoing MV in our medical ICU between October 2002, and June 2003. Study exclusion criteria were age<18 years, duration of MV<24 hours (to exclude those who required a short course of intubation for overdose), tracheostomy at the time of initiation of MV, transfer from another ICU service, location other than our medical ICU, or prisoners. If patients had multiple courses of MV during their hospitalization, only the first episode was evaluated. Sedation was managed according to our medical ICU algorithm and based on published recommendations [5,15]. The sedation algorithm goals were to maximize the use of boluses, to minimize the duration of continuous intravenous infusion of sedation, and to treat pain with opioids. Weaning from MV was also standardized through the use of daily spontaneous breathing trials and was guided by bedside Nurses, Respiratory Therapists and Physicians [16].

AOD was classified according to the substance used: alcohol, benzodiazepines or barbiturates, heroin, opioids (other than heroin), cocaine, amphetamines, marijuana and other (excluding nicotine). Diagnosis of AOD was assigned if the disorder was present within one year previous to initiation of MV. Medical records were reviewed for the target admission as well as previous admissions and outpatient visits. To maximize diagnostic accuracy, experts in Addiction Medicine and Psychiatry assigned the diagnosis (MFW, JT). Diagnosis was established through review of toxicology results, medical records, and based on the Diagnostic and Statistical Manual of Mental Disorders, 4^th ^Edition TR (DSM IV-TR) (Table 1) [17].

**Table 1:** Definition of Substance Dependence and Substance Abuse

Substance dependence is manifested by three or more of the following:
(i) tolerance, as marked by the need for larger doses to achieve intoxication or desired effect or markedly diminished effect with continued use of the same amount of substance;
(ii) development of withdrawal symptoms or use of substance to relieve or avoid withdrawal symptoms;
(iii) taking larger amounts or over longer periods than intended;
(iv) persistent desire or unsuccessful efforts to cut down or control substance use;
(v) spending time obtaining the substance, using the substance or recovering from its effects;
(vi) performing important social, occupational or recreational activities less frequently because of the substance;
(vii) continuing use of the substance despite knowledge of adverse physical or psychological problems.

Substance abuse is manifested by at least one of the following:

(i) recurrent use resulting in failure to fulfill major obligations at work, home or school;
(ii) recurrent use in situations in which it is physically hazardous;
(iii) recurrent substance-related legal problems;
(iv) continued use despite having persistent or recurrent social or interpersonal problems.

• Medical record: When physician notes explicitly documented the presence of alcohol or drug abuse, dependence, and/or addiction, the patient was classified as having AOD. When the terms abuse, dependence or addiction were not documented in the record, the patient was not diagnosed with AOD based on review of medical records alone.

• Clinical diagnosis: Definitions of substance dependence and substance abuse as defined in DSM IV-TR are outlined in Table 1. If details in the medical history permitted, patients were diagnosed with AOD. For example, patients who presented to the Emergency Department with trauma and intoxication were diagnosed with AOD. As another example, patients with a history of withdrawal syndromes while on the inpatient ward were diagnosed with AOD. As a third example, patients with history of alcoholic cirrhosis who had consumed alcohol within the previous year were diagnosed with AOD.

• Toxicology results: If these revealed the presence of cocaine, heroin, marijuana, or amphetamine, patients were considered to have AOD. Patients with toxicology results that were positive for other opioids, benzodiazepines, or barbiturates were diagnosed with AOD if the substances were not administered by a healthcare professional prior to collection of urine or blood samples. Positive toxicology results for alcohol could be used only if they supported a clinical diagnosis (e.g. alcohol withdrawal syndromes, alcoholic cirrhosis), and could not be used in isolation.

Investigators who collected the remaining data (MdW, SYW, SG, WIJ) were blinded to the diagnosis of AOD. Similarly, Addiction Medicine (MFW, JT) experts were blinded to the remaining data.

Baseline characteristics of age, gender, race, ethnicity, reason for MV (which is also the reason for ICU admission and the major diagnosis), ratio of partial pressure of oxygen to fraction of inspired oxygen (P/F) positive end expiratory pressure (PEEP), and Acute Physiology Chronic Health Evaluation II (APACHE II) were collected [18]. Because AOD patients may present with altered mentation, we adjusted APACHE II for Glasgow Coma Score (GCS; adjusted APACHE II = APACHE II - (15 - GCS)) [19]. Sequential Organ Failure Assessment (SOFA) was computed for the first day of MV and was also adjusted for GCS (adjusted SOFA = Respiratory score + Platelet score + Bilirubin score + Hypotension score + Renal score) [20]. The total amount of sedatives and opioids administered during the course of MV was calculated, and duration of continuous intravenous infusion was recorded. Benzodiazepines and barbiturates were converted to lorazepam equivalents, and opioids were converted to morphine equivalents using referenced conversion formulas [21,22]. Dose of propofol was also recorded and not converted to lorazepam equivalents because of unavailability of published data. We limited analysis to sedative and opioid administration with abuse potential and therefore did not record administration butyrophenones and phenothiazines. Sedation depth using Richmond Agitation-Sedation Scale (RASS, Table 2), as routinely recorded by nursing staff every 4 hours, was collected [23]. Duration of MV, reintubation within 72 hours of extubation, unplanned extubation, placement of tracheostomy, ICU mortality, hospital mortality, hospital length of stay and ICU length of stay were recorded.

**Table 2:** Richmond Agitation-Sedation Scale

**Score**	**Term**	**Description**
+4	Combative	Overtly combative or violent. Immediate danger to staff
+3	Very agitated	Pulls on or removes tube(s) or catheter(s), or has aggressive behavior toward staff
+2	Agitated	Frequent nonpurposeful movement or patient ventilator dyssynchrony
+1	Restless	Anxious or apprehensive but movements not aggressive or vigorous
0	Alert and calm	
-1	Drowsy	Not fully alert, but has sustained (>10 seconds) awakenings, with eye contact, to voice
-2	Light sedation	Briefly (<10 seconds) awakens with eye contact to voice
-3	Moderate sedation	Any movement (but no eye contact) to voice
-4	Deep sedation	No response to voice, but any movement to physical stimuli
-5	Unarousable	No response to voice or physical stimulation

### Data analysis

The primary aim compares patients with AOD to those without AOD. Duration of MV was computed using Kaplan-Meier method and compared by log rank. Normally distributed data were compared using two-group t-test. Non-normally distributed data were compared using Wilcoxon Test. A mixed-model repeated-measures ANOVA was used to compare sedation levels. The prevalence of AOD in the study was compared to that of the city of Richmond, Virginia (17.9%), using Chi-square [24]. ICU and hospital mortality rates as well as reintubation and tracheostomy rates were compared using Chi-square or Fisher's Exact test when appropriate. ICU and hospital length of stay were compared using log rank. Alpha was set at 0.05. Normally distributed data are reported as mean and 95% confidence interval (CI), and non-normally distributed data as median and interquartile range (IQR), or median and 95% CI.

### Sample size calculation

We had a priori estimated the proportion of patients with AOD to be 30–40%. Study primary endpoint was total dose of sedative and total dose of opioids administered. Power analysis indicated that with 70 patients, we would be able to detect a 0.7 standard deviation difference with 80% power and a 0.85 standard deviation with 90% power.

## Results

Three hundred fifty-three patients requiring MV were admitted to the medical ICU between October 2002 and June 2003. One hundred forty-nine patients selected by the random number generation algorithm were screened. Seventy-nine patients failed to meet the pre-specified inclusion criteria for the following reasons: age<18 years (1), duration of MV<24 hours (22), tracheostomy at the time of initiation of MV (2), transfer from another ICU (20), location other than medical ICU (24), prisoner (10).

**Figure 1:**
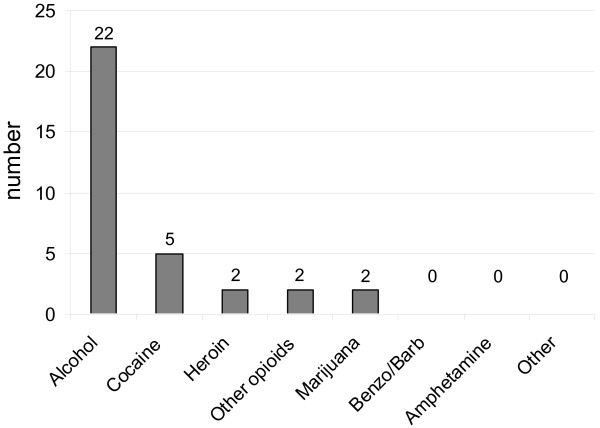
Distribution of substances implicated in alcohol and other drug use disorders. Benzo/Barb: benzodiazepines or barbiturates.

Of the 70 patients meeting the inclusion criteria, 27 (39%) were diagnosed with AOD (Figure 1). The diagnosis of AOD was established based on toxicology results in 5 (19%), identification by the healthcare provider in the medical record in 16 (59%), and by the DSM IV-TR criteria in 7 (26%) patients. Of the 21 patients who underwent toxicology screens, 1 was positive for alcohol, 4 for cocaine, 5 for benzodiazepines, and 7 for opiates. All positive benzodiazepine and opiate screens could be accounted for by administration of medication prior to sample acquisition. No toxicology screen was positive for amphetamine, barbiturate, or cannabis.

Overall, alcohol was implicated in 22 cases (31%) and other drugs in 7 (10%). Of the 22 patients with alcohol use disorders, alcohol was the only implicated substance in 20 (91%) cases, while 2 patients had other drug use disorders (marijuana in 1 case and cocaine, opioids and marijuana in the second case). Of the 7 patients with other drug use disorders, heroin and/or cocaine were implicated in 5 patients. When comparing the prevalence of AOD in our medical ICU patients (39%) to the population prevalence of the city of Richmond (17.9%), the medical ICU rate was significantly higher (p < 0.0001).

Baseline characteristics for patients are outlined in Table 3. Patients with AOD were more likely to be male. Although one patient with AOD was intubated for delirium tremens, analyses revealed no difference in reason for MV between the two groups. Patients with AOD were less severely ill as measured by APACHE II, and adjustment of GCS suggested a trend toward lower severity of illness (p = 0.07). The two groups had similar Sequential Organ Failure Assessment (SOFA) scores on the first day of MV and adjusting for GCS did not change the results.

**Table 3:** Baseline Characteristics

	**AOD**	**No AOD**	**p**
n	27	43	
Age (years) (mean [95% CI])	50 [45.0; 55.8]	55 [50.6; 59.2]	0.20
Gender (n men/women)	21/6	15/28	<0.0001
Race (n African American/White/Asian)	20/7/0	22/19/2	0.12
APACHE II (mean [95% CI])	22 [18.2; 25.2]	26 [23.5; 29.0]	0.048
APACHE II excluding GCS (mean [95% CI])	13 [10.6; 17.1]	18 [15.1; 20.2]	0.07
SOFA (mean [95% CI])	8 [6.3; 9.3]	9 [7.5; 9.9]	0.33
Bilirubin* (mg/dl) (mean [95% CI])	2.2 [0.65; 3.85]	1.8 [0.52; 3.07]	0.65
Creatinine (mg/dl) (median, IQR)	1.1 [0.80; 2.20]	1.5 [1.10; 2.80]	0.10
P/F (mean [95% CI])	225 [177.4; 272.8]	218 [181.5; 254.2]	0.81
PEEP (cm H_2_O) (mean [95% CI])	5 [4.4; 6.4]	5 [3.9; 5.6]	0.30
Reason for mechanical ventilation			0.46
Pneumonia/ALI	4	9	
Sepsis	1	4	
Delirium/Neurologic	7	10	
Asthma/COPD	3	4	
Upper airway obstruction	4	3	
Hemorrhagic shock	3	4	
Cardiac	1	4	
Cardiopulmonary arrest	1	2	
Drug overdose	3	0	
Other	0	3	

AOD: alcohol and other drug use disorders

CI: confidence interval

APACHE II: Acute Physiology and Chronic Health Evaluation II

GCS: Glasgow Coma Score

SOFA: Sequential Organ Failure Assessment

*Bilirubin: Total bilirubin measured in 14 patients with AOD and 22 patients without AOD

IQR: Interquartile range

P/F: Partial pressure of oxygen divided by fraction of inspired oxygen

PEEP: Positive end expiratory pressure

Patients with AOD were significantly more likely to receive benzodiazepines (27 out of 27 patients versus 39 out of 43, p = 0.044) and opioids (26 out of 27 patients versus 31 out of 43, p = 0.006) compared to patients without AOD. Administered opioids included morphine (AOD 15 versus non-AOD 21, p = 0.38), fentanyl (AOD 13 versus non-AOD 16, p = 0.26), hydromorphone (AOD 3 versus non-AOD 0, p = 0.22), and meperidine (AOD 0 versus non-AOD 1, p = 0.39). There was a trend toward increase methadone administration in the AOD group (AOD 4 versus non-AOD 1, p = 0.07). The likelihood of receiving propofol was similar in the two groups (12 out of 27 versus 21 out of 43, p = 0.72). Table 4 summarizes the administered sedative and opioid doses. The group of patients with AOD received 2.5 times and 5 times the total doses of benzodiazepines and opioids, respectively, compared to the group without AOD. Propofol dose did not differ in the two groups. While the number of continuously infused sedatives and opioids was similar for AOD and non-AOD patients (2.1 versus 1.8, p = 0.15), the mean infusion duration in the AOD group was longer (16 hours/day.medication, 95%CI [12.7; 18.3] versus 10 [7.6; 12.1], p = 0.002).

**Table 4:** Sedative and Opioid Doses

	**AOD**		**No AOD**		**p**
	
	Median	IQR	Median	IQR	
Lorazepam equivalents (mg/kg.day)	0.5	0.32–1.08	0.2	0.02–0.63	0.004
Morphine equivalents (mg/kg.day)	0.5	0.03–2.68	0.1	0.00–0.93	0.03
Propofol (microg/kg.day)*	0	0–28	0	0–14	0.81

AOD: alcohol and other drug use disorders

IQR: interquartile range

*33 patients received propofol, of which 12 had AOD

A total of 2381 RASS values were recorded during 362 days of MV for the 70 study patients. The number of assessments was similar between patients with and without AOD (28 observations, IQR [16.0; 39.0] versus 30, IQR [14.0; 40.2], p = 0.89). The mean RASS was similar among AOD patients (-2; 95% CI [-2.6; -1.6]) and non-AOD patients (-2; 95% CI [-2.4; -1.6], p = 0.83). However, AOD patients had a larger variance in RASS (3 RASS units, 95%CI [2.3; 3.7] versus 2, 95%CI [1.5; 2.6], p = 0.049), indicating larger fluctuations in sedation levels. Patients with AOD were not more frequently agitated as measured by RASS ≥ 3 compared to those without AOD (3.0% of observations, 95%CI [2.1; 4.2]% versus 2.4%, 95%CI [1.8; 3.2]%, p = 0.33).

**Figure 2:**
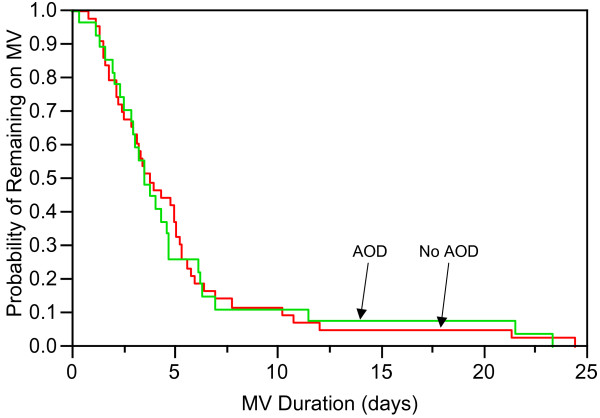
Kaplan-Meier estimate of probability of remaining on mechanical ventilation (MV) in patients with alcohol and other drug use disorders (AOD) and those without (No AOD).

AOD patients and non-AOD patients had similar MV duration (3.6 days, 95%CI [2.60; 4.71] versus 3.9, 95%CI [2.91; 5.14], p = 0.89) (Figure 2). Adjustment for gender, APACHE II, lorazepam equivalents, morphine equivalents, and duration of continuous infusions did not change the results. Because mortality on MV may be more a measure of time until death than outcome of mechanical ventilation management, we analyzed MV duration for ICU survivors and non-survivors. Twenty patients died in the ICU, 6 with AOD and 14 without AOD. The median duration of MV in non-survivors with AOD tended to be shorter compared to ICU non-survivors without AOD (AOD 3.6 days, 95% CI [0.43; undeterminable] versus non-AOD 5.4 days, 95% CI [2.22; 10.25], p = 0.12). Severity of illness in the two groups was similar as measured by APACHE II (AOD 32, 95% CI [23.7; 39.7] versus non-AOD 31, 95% CI [26.0; 36.4], p = 0.92) and SOFA (AOD 12, 95% CI [8.0; 15.0] versus non-AOD 11, 95% CI [8.3; 12.9], p = 0.65). Adjustment of APACHE II for GCS (AOD 23, 95% CI [15.6; 30.8] versus non-AOD 23, 95% CI [17.5; 27.5], p = 0.88) and adjustment of SOFA for GCS (AOD 8, 95% CI [5.4, 11.6] versus non-AOD 7, 95% CI [5.4; 9.5], p = 0.55) did not change severity of illness.

Fifty patients survived the ICU, 21 with AOD and 29 without AOD. Duration of MV for ICU survivors was similar for the two groups (AOD 3.8 days, 95% CI [2.36; 4.73] versus non-AOD 3.4 days, 95% CI [2.35; 4.82], p = 0.87). AOD patients who survived were less severely ill as measured by APACHE II (AOD 19, 95% CI [15.5; 22.3] versus non-AOD 24, 95% CI [21.0; 26.7] p = 0.03) but not SOFA (AOD 7, 95% CI [5.2; 8.2] versus non-AOD 8, 95% CI [6.6; 9.1], p = 0.25). APACHE II adjusted for GCS remained lower in the AOD group (AOD 11, 95% CI [8.2; 14.3] versus non-AOD 15, 95% CI [12.7; 17.9], p = 0.045). SOFA adjusted for GCS remained similar for the AOD and non-AOD group (AOD 5, 95% CI [3.7; 5.8] versus non-AOD 4, 95% CI [2.8; 5.3], p = 0.40).

There was no difference in the rate of reintubation (11% versus 9%, p = 0.81), tracheostomy (7% versus 2%, p = 0.31), or hospital mortality (22% versus 40%, p = 0.13). ICU length of stay (6 days [3.4; 6.9] versus 6 days, 95% CI [4.4; 6.8], p = 0.91) and hospital length of stay (9 days, 95%CI [5.3; 11.9] versus 13 days, 95%CI [9.7; 18.2], p = 0.11) were similar between patients with and without AOD. There were no unplanned extubations.

## Discussion

The current study reveals that nearly 40% of all mechanically ventilated patients in our medical ICU suffer from alcohol or other drug use disorders, with alcohol predominating. Additionally, our study found that AOD patients receive a greater amount of sedatives and opioids than their non-AOD comparators in order to achieve a similar degree of sedation. Despite this greater exposure to sedatives, the duration of MV was similar in the AOD and the non-AOD groups.

AOD is a common problem in our medical ICU, affecting 27 out of 70 (39%) of our long-term mechanically ventilated patients. The true rate in our patient population is likely to be even higher because of the retrospective nature of our study and because clinicians fail to diagnose AOD in 10% to 82% of patients [25,26]. Our rate is substantially higher than that reported in the literature and may be explained by methodological issues. Other studies have examined rates of admissions directly attributable to AOD or have limited diagnosis to alcohol use disorders [9,10]. We included all patients where AOD was either a primary or other diagnosis. The prevalence of AOD in our mechanically ventilated patients was significantly higher than that in the surrounding community, indicating that patients with AOD are at increased risk of requiring MV. This finding has also been demonstrated by others. Moss et al. have shown that patients with alcohol use disorders and sepsis are more likely to require MV compared to septic patients without alcohol use disorders [10]. Saitz et al. have shown that patients with pneumonia who have alcohol use disorders are at increased risk of requiring ICU level care, and Suchyta et al. have shown that patients with AOD and other psychiatric disorders are overrepresented among ICU patients [11,27].

Alcohol was the most commonly implicated substance, which is similar to national findings, and our rate of 31% among MV patients is similar to the 30% reported in a prospective study by Moss et al [10]. Also consistent with national findings, in 91% of all patients alcohol use disorders, alcohol was the only substance implicated [8]. Among non-alcohol drug use disorders, cocaine and heroin, the two most common illicit drugs used in the city of Richmond, were the most commonly implicated substances in our patient population (5 out of 7 patients) [24]. Not surprisingly, no patient was diagnosed with amphetamine use disorders, since these substances are more commonly used in the Western part of the United States than Virginia [28]. Additionally, the highest prevalence of amphetamine use disorders is in the age group 18–34 years old, which is generally younger than our study population.

Patients with AOD required 2.5 times more sedative and 5 times more opioid doses to achieve sedation levels similar to patients without AOD. They also received longer duration of infusions which likely resulted in higher plasma levels and suggest a high degree of tolerance [29]. Although no difference was seen in propofol dose, relatively few patients received this sedative. Since sedation was managed according to a standardized algorithm in our ICU, it is not likely that the higher dosages seen in the AOD patients were driven by a bias towards greater levels of sedation in this population, but rather by the true need to achieve a pre-specified level of sedation. This is further supported by tolerance and increased metabolism through induction of the cytochrome P-450 enzyme system documented previously in this population [30]. Patients with AOD and those without AOD have similar bilirubin and creatinine, suggesting that lower doses in non-AOD patients are not accounted for by impaired metabolism and clearance. Additionally, P/F and PEEP were similar for both groups, suggesting no difference in lung injury and need for sedation.

Patients with AOD had duration of MV similar to patients without AOD. Patients without AOD had a higher number of patients with pneumonia, acute lung injury and sepsis, diagnoses that are associated with longer duration of mechanical ventilation [31]. It is possible that this could have lead to a longer MV duration in the group of patients without AOD. Additionally, patients without AOD had higher severity of illness which may also have resulted in longer duration of mechanical ventilation compared to the group with AOD. It is conceivable that patients with AOD did not have a shorter MV duration because of the increased sedative and opioid requirement. The longer expected MV duration in the group of patients without AOD may have been eliminated by the increased sedative and opioid requirements in patients with AOD, resulting is similar MV duration in the 2 groups.

Our study has several strengths. The study was conducted at a large urban medical center, and patients were randomly selected. Diagnoses were established by clinical experts in Addiction Medicine who were blinded to the amount of administered sedatives and opioids and duration of MV. To minimize the bias in assigning an AOD diagnosis retrospectively, toxicology data and healthcare provider history were used whenever possible; when not available, very strict adherence to the definitions of the DSM IV-TR was established. Both sedation and weaning from MV are standardized in our ICU, thus eliminating the potential confounding effect of differential preference-based practices in this area on the outcomes of interest.

Our study has limitations. The diagnosis of AOD is difficult to establish in patients, and this is particularly problematic in non-verbal critically ill patients undergoing MV. Screening for AOD is not standardized in our ICU and is at the discretion of clinicians. In our experience, intensivists do not routinely determine the presence of these disorders in their patients. The definition of AOD is broad and includes behavioral and social aspects, and clinicians may focus on the aspects of physiologic dependence, tolerance and withdrawal during critical illness. Additionally, next-of-kin may not be forthcoming with information about AOD. These factors contribute to the underdiagnosis and misclassification of some study patients. Despite this limitation, we were able to determine significant differences between patients assigned a diagnosis of AOD and those not assigned this diagnosis, indicating that patients with AOD are different from those without AOD. The study sample was small; however, even the small number of charts reviewed had sufficient power to detect significant differences in the primary outcome, again supporting the findings that patients with AOD are quite different from their non-AOD counterparts. The study was limited to a single center's medical ICU, excluding patients in the surgical ICU, patients with primarily cardiac diagnoses, and trauma patients which may limit its generalizability.

## Conclusion

Our study is the first to identify AOD as an important comorbidity that impacts sedation management while on MV. AOD patients require a greater amount of sedatives and opioids to achieve the same level of sedation. ICU clinicians need to be cognizant of the potential influence of AOD on the course and management of their mechanically ventilated patients, particularly in those ICU's that do not utilize a clinical practice guideline-driven sedation protocol, in order to avoid potential complications associated with over- or undersedation. Given a problem of such an extensive magnitude, AOD among MV patients needs to be studied further in prospective studies to gain a better understanding of how to improve sedation and other outcomes in these patients.

## Abbreviations

ANOVA  - Analysis of variance

APACHE II - Acute Physiology and Chronic Health Evaluation II

AOD - Alcohol and other drug use disorders

Benzo/Barb - Benzodiazepines or barbiturates

CI - Confidence interval

DSM IV-TR -  Diagnostic and Statistical Manual of Mental Disorders, 4^th ^Edition TR

GCS - Glasgow Coma Score

ICU - Intensive care unit

IQR - Interquartile range

MV - Mechanical ventilation

P/F - Partial pressure of oxygen divided by fraction of inspired oxygen

PEEP - Positive end expiratory pressure

RASS - Richmond Agitation-Sedation Scale

SOFA - Sequential Organ Failure Assessment

## Competing interests

The authors declare that they have no competing interests. The study was funded by NIH K23 GM068842 and NIH M01 RR00065.

## Authors' contributions

MdW participated in study design, data acquisition, data analysis, data interpretation, and manuscript preparation. SYW, SG, and WIJ participated in data acquisition, data interpretation, and manuscript preparation. AMB participated in study design, data analysis, data interpretation, and manuscript preparation. JT and MFW participated in study design, data acquisition, data interpretation, and manuscript preparation. All authors have given final approval to manuscript.

## Pre-publication history

The pre-publication history for this paper can be accessed here:


